# A High Grade of Postoperative Knee Laxity Is Associated With an Increased Hazard of Revision Surgery: A Cohort Study of 4697 Patients With Primary ACL Reconstruction

**DOI:** 10.1177/03635465241253840

**Published:** 2024-05-31

**Authors:** Riccardo Cristiani, Magnus Forssblad, Camilo P. Helito, Gunnar Edman, Karl Eriksson, Anders Stålman

**Affiliations:** † Department of Molecular Medicine and Surgery, Stockholm Sports Trauma Research Center, Karolinska Institutet, Stockholm, Sweden; ‡ Capio Artro Clinic, FIFA Medical Centre of Excellence, Stockholm, Sweden; § Grupo de Joelho, Instituto de Ortopedia e Traumatologia, Hospital das Clínicas HCFMUSP, Faculdade de Medicina da Universidade de São Paulo, São Paulo, Brazil; ‖ Hospital Sírio Libanês, São Paulo, Brazil; ¶ Department of Orthopaedics, Stockholm South Hospital, Stockholm, Sweden; # Karolinska Institutet, Stockholm, Sweden; Investigation performed at Capio Artro Clinic/Stockholm Sports Trauma Research Center, Karolinska Institutet, Stockholm, Sweden

**Keywords:** ACL reconstruction, knee laxity, patient-reported outcome, revision ACL reconstruction

## Abstract

**Background:**

There is still debate regarding the association between arthrometric knee laxity measurements and subjective knee outcome and revision surgery after primary anterior cruciate ligament reconstruction (ACLR).

**Purpose:**

To assess whether arthrometric knee laxity (measured with the KT-1000 arthrometer) 6 months after primary ACLR was associated with the 1-, 2-, and 5-year subjective knee outcomes or revision ACLR at a 5-year follow-up.

**Study Design:**

Cohort study, Level of evidence 3.

**Methods:**

Patients who underwent primary ACLR with a hamstring tendon autograft at the authors’ institution between January 1, 2005, and December 31, 2017, with no concomitant ligamentous injuries, were identified. Anterior knee laxity (KT-1000 arthrometer, 134 N) was assessed 6 months postoperatively. The Knee injury and Osteoarthritis Outcome Score (KOOS) was collected preoperatively and 1, 2, and 5 years postoperatively. Patients who underwent revision ACLR at any institution in the country within 5 years of primary surgery were identified through the Swedish National Knee Ligament Registry.

**Results:**

A total of 4697 patients (54.3% male) with available KT-1000 arthrometer measurements were included (normal: side-to-side [STS] ≤2 mm, 3015 [64.2%]; nearly normal: STS 3-5 mm, 1446 [30.8%]; abnormal: STS >5 mm, 236 [5.0%]). The only significant difference in subjective knee outcome between the groups was for the KOOS Symptoms subscale at the 1-year follow-up (STS ≤2 mm, 79.9 ± 16.2; STS 3-5 mm, 82.5 ± 14.8; STS >5 mm, 85.1 ± 14.2; *P* < .001). No other significant differences between the groups were found preoperatively or at 1, 2, or 5 years postoperatively for any of the KOOS subscales. The hazard for revision ACLR within 5 years of the primary surgery was significantly higher for the groups with an STS of 3 to 5 mm (6.6%; 95/1446) (hazard ratio [HR], 1.42; 95% CI, 1.07-1.87; *P* = .01) and an STS >5 mm (11.4%; 27/236) (HR, 2.61; 95% CI, 1.69-4.03; *P* < .001) compared with the group with an STS ≤2 mm (3.8%; 116/3015).

**Conclusion:**

A high grade of postoperative knee laxity (STS 3-5 mm and STS >5 mm) 6 months after primary ACLR was associated with an increased hazard of revision ACLR within 5 years, but it was not associated with an inferior subjective knee outcome.

The anterior cruciate ligament (ACL) is the primary restraint to anterior translation and internal rotation of the tibia.^5,21^ From a surgical perspective, the main goal of ACL reconstruction (ACLR) is to restore knee laxity to a level as close as possible to that of the native (ACL-intact) knee. The currently most used instrument to evaluate anterior knee laxity is the KT-1000 arthrometer (MEDmetric).^7,24^ This instrument has proven to be reliable^14,20,25,42^ and is suggested to be more precise than the clinical (Lachman and anterior drawer) tests,^13,41^ as it gives a quantitative evaluation of the anterior tibial displacement. The KT-1000 arthrometer is used not only to diagnose an ACL injury, but also after surgery to measure the restraint provided by the ACL graft.^7,11,13^ According to the International Knee Documentation Committee (IKDC) form,^19^ a side-to-side (STS) difference ≤2 mm is considered to be normal, whereas STS differences between 3 and 5 mm and >5 mm are considered to be nearly normal and abnormal, respectively. Nonetheless, questions remain about how residual postoperative arthrometric knee laxity affects subjective knee outcome or the risk of revision ACLR. The few existing studies have yielded mixed results. Some authors^16,24^ have reported that residual postoperative arthrometric knee laxity is associated with inferior subjective knee outcome and increased risk of revision ACLR, whereas others^18,35^ have reported no association.

The purpose of this study was to assess whether arthrometric knee laxity 6 months after primary ACLR was associated with 1-, 2-, and 5-year subjective knee outcomes or the risk of revision ACLR at a 5-year follow-up. We hypothesized that a greater postoperative knee laxity would be associated with an inferior subjective knee outcome and a higher risk of revision ACLR.

## Methods

### Participants

Ethical approval for this study was obtained from the regional ethics committee, Karolinska Institutet (Diarienumber 2016/1613-31/32).

Patients who underwent primary ACLR with a hamstring tendon autograft at our institution between January 1, 2005, and December 31, 2017, with no concomitant ligament injuries, were assessed for eligibility. Patients with a contralateral ACL injury or reconstruction and with missing postoperative arthrometric measurements were excluded.

### Surgical Technique and Rehabilitation

A single-bundle technique was used for all cases. The semitendinosus tendon was primarily harvested and prepared as a tripled or quadrupled graft. In the event of an insufficient length or diameter (<8 mm), the gracilis tendon was harvested and combined with the semitendinosus tendon graft. An anteromedial portal technique was used to drill the femoral tunnel. The graft was routinely fixed with an Endobutton fixation device (Smith & Nephew) on the femoral side and Ethibond No. 2 sutures (Ethicon) tied over an AO bicortical screw with a washer as a post or with an interference screw on the tibial side. An arthroscopic all-inside technique using Fast-Fix suture anchor devices (Smith & Nephew) was used to repair meniscal tears located in the posterior horn and corpus of the menisci. Meniscal tears located in the anterior portion of the menisci were repaired via an outside-in technique using PDS 0 (Ethicon). The patients followed a supervised and standardized postoperative rehabilitation protocol. Full weightbearing and range of motion were allowed in the event of an isolated ACLR or ACLR and concomitant meniscal resection. In the event of an ACLR and concomitant meniscal repair, the patients wore a hinged knee brace for 6 weeks. The brace was set to allow for 0° to 30° of flexion for the first 2 weeks, 0° to 60° of flexion for the third and fourth weeks, and 0° to 90° for the fifth and sixth weeks postoperatively. Starting from the seventh week, the brace was discontinued, and full range of motion was encouraged. Quadriceps strengthening was restricted to closed kinetic chain exercises during the first 3 months. The patients were allowed to return to sports 6 months after ACLR at the earliest if the isokinetic knee muscle strength and single-leg hop test criteria (limb symmetry index, ≥90%) were met.^9,39^

### Arthrometric Evaluation

Instrumented knee laxity was assessed 6 months postoperatively at our outpatient clinic. The KT-1000 arthrometer with a standard anterior tibial load of 30 pounds (134 N) at 20° of knee flexion was used. All laxity assessments were performed by experienced sports medicine physical therapists. At least 3 measurements of each knee were made, and the median value was registered. The STS difference (injured knee – healthy knee) was registered. STS knee laxity was stratified and classified into 3 groups (≤2 mm, 3-5 mm, and >5 mm) according to the IKDC examination form.^19^

### Subjective Knee Outcome

The Knee injury and Osteoarthritis Outcome Score (KOOS)^32,33^ was used to evaluate subjective knee function preoperatively and at 1, 2, and 5 years postoperatively. The KOOS consists of 5 subscales: Symptoms, Pain, Activities of Daily Living, Sport and Recreation, and knee-related Quality of Life. The scores are reported as a number for each subscale, with 0 representing the worst possible outcome and 100 the maximum score.

### Revision ACLR

Patients who underwent revision ACLR within 5 years of primary surgery at any institution in the country between January 1, 2005, and December 31, 2022, were identified using their unique Swedish personal identity number^26^ in the Swedish National Knee Ligament Registry (SNKLR).^37^

### Data Sources

Data collected from our clinic database were age at primary ACLR, time from injury to primary ACLR, sex, the presence of a cartilage injury, the preinjury Tegner activity level,^38^ graft diameter, and meniscal surgery (classified as medial meniscal or lateral meniscal resection or repair). Arthrometric (KT-1000 arthrometer) laxity measurements were also collected from our clinic database. Finally, the KOOS values were collected from the SNKLR.^37^

### Statistical Analysis

The Statistical Product and Service Solutions (SPSS) (Version 25.0; IBM Corp) was used for the statistical analysis. The variables were summarized with standard descriptive statistics, such as mean ± SD or frequency. The distributions were checked for deviation from a normal distribution. An analysis of variance and Pearson chi-square tests were used for comparisons of continuous and categorical variables, respectively. Preoperative and 1-, 2-, and 5-year postoperative KOOS subscale values were compared among the laxity groups using an analysis of covariance, with age at primary ACLR, sex (preoperative and postoperative KOOS values), and medial meniscal resection (postoperative KOOS) as covariates. These covariates were selected because there were differences between the groups in these variables, and previous literature has shown that these factors may potentially affect the KOOS subscale values before and after ACLR.^1,8,15^ A Cox regression analysis was used to compare the 5-year revision risk between the laxity groups. The laxity group with an STS laxity ≤2 mm was used as a reference group. Hazard ratios (HRs) were adjusted for age at primary ACLR, sex, time from injury to primary ACLR, and medial meniscal resection. These confounders were added to the model because there were differences between the laxity groups, and previous studies have shown that these factors may potentially affect the risk of revision ACLR.^6,31,36^ The level of significance in the analyses was 5% (2-tailed).

## Results

A total of 6739 patients were reviewed for eligibility. After excluding patients with a contralateral ACL injury or reconstruction (n = 489) and with no available postoperative KT-1000 arthrometer measurements (n = 1553), 4697 patients were eligible. The patients were then divided in 3 groups depending on postoperative KT-1000 arthrometer values according to the IKDC knee examination form: ≤2, 3 to 5, and >5 mm. The patients’ characteristics are summarized in Table 1.

**Table 1:** Patient Characteristics^*a*^

	STS ≤2 mm	STS 3-5 mm	STS >5 mm	*P*
No. of patients	3015 (64.2)	1446 (30.8)	236 (5.0)	
STS difference, mm	0.6 ± 1.4	3.7 ± 0.7	6.8 ± 1.1	<.001^*b*^
Age at surgery, y	29.3 ± 10.8	27.6 ± 10.6	27.1 ± 10.8	<.001^*b*^
Time from injury to surgery, mo	16.1 ± 30.4	15.6 ± 26.4	22.6 ± 35.1	.004^*b*^
Patients with available data, n	2805	1363	221	
Sex				
Male	1680 (55.7)	748 (51.7)	127 (53.8)	.04^*b*^
Female	1335 (44.3)	698 (48.3)	109 (46.2)	
Cartilage injury	548 (18.2)	264 (18.3)	38 (16.1)	.71
MM resection	406 (13.5)	247 (17.1)	69 (29.2)	<.001^*b*^
MM repair	176 (5.8)	102 (7.1)	12 (5.1)	.22
LM resection	476 (15.8)	252 (17.4)	34 (14.4)	.27
LM repair	108 (3.6)	58 (4.0)	11 (4.7)	.59
Preinjury Tegner score	7.2 ± 1.7	7.2 ± 1.7	7.0 ± 1.7	.34
Patients with available data, n	2745	1308	203	
Graft diameter, mm	8.4 ± 0.8	8.3 ± 0.8	8.3 ± 0.7	.65
Patients with available data, n	2485	1120	209	

a Data are reported as n (%) or mean ± SD unless otherwise indicated. LM, lateral meniscus; MM, medial meniscus; STS, side to side.

b Statistically significant.

### Subjective Knee Outcome

The only significant difference between the groups was seen in the KOOS Symptoms subscale at the 1-year follow-up (STS ≤2 mm, 79.9 ± 16.2; STS 3-5 mm, 82.5 ± 14.8; STS >5 mm, 85.1 ± 14.2; *P* < .001). No other significant differences between the groups were found preoperatively or at 1, 2, or 5 years postoperatively for any of the KOOS subscales (Table 2).

**Table 2:** Preoperative and Postoperative KOOS Comparison^*a*^

	STS ≤2 mm	STS 3-5 mm	STS >5 mm	*P*
Symptoms				
Preoperative	2712 (75.5 ± 17.1)	1369 (76.1 ± 17.3)	220 (75.4 ± 18.6)	.51
1 y	2267 (79.9 ± 16.2)	1154 (82.5 ± 14.8)	177 (85.1 ± 14.2)	<.001^*b*^
2 y	1590 (82.4 ± 15.7)	823 (83.8 ± 15.4)	130 (82.4 ± 16.3)	.09
5 y	1400 (84.7 ± 15.6)	682 (84.2 ± 15.9)	99 (86.4 ± 15.0)	.41
Pain				
Preoperative	2712 (78.1 ± 16.7)	1368 (79.9 ± 15.9)	220 (79.4 ± 16.1)	.76
1 y	2268 (88.0 ± 12.2)	1154 (89.0 ± 11.9)	176 (88.4 ± 12.1)	.05
2 y	1590 (89.0 ± 12.6)	823 (89.0 ± 13.5)	130 (87.1 ± 16.5)	.27
5 y	1400 (89.9 ± 12.8)	682 (89.1 ± 13.4)	99 (89.9 ± 14.2)	.41
ADL				
Preop	2710 (88.0 ± 13.8)	1369 (88.2 ± 14.4)	220 (86.7 ± 15.2)	.35
1 y	2268 (94.4 ± 9.3)	1154 (94.7 ± 9.5)	177 (94.4 ± 9.2)	.66
2 y	1590 (94.3 ± 10.3)	823 (94.3 ± 11.1)	130 (92.5 ± 13.6)	.16
5 y	1400 (94.8 ± 9.8)	682 (94.6 ± 9.9)	99 (94.3 ± 11.5)	.78
Sport and Recreation				
Preoperative	2580 (50.8 ± 26.8)	1300 (51.7 ± 26.7)	208 (51.1 ± 28.8)	.58
1 y	2245 (72.1 ± 22.8)	1141 (72.8 ± 22.6)	176 (72.8 ± 24.4)	.63
2 y	1571 (74.5 ± 22.7)	818 (75.6 ± 23.4)	128 (71.7 ± 28.9)	.17
5 y	1389 (75.4 ± 24.0)	674 (75.8 ± 23.6)	96 (78.3 ± 24.9)	.52
QOL				
Preoperative	2651 (38.7 ± 22.0)	1337 (38.9 ± 22.1)	213 (39.4 ± 23.1)	.91
1 y	2260 (62.5 ± 22.0)	1147 (63.1 ± 22.2)	176 (63.1 ± 23.9)	.72
2 y	1585 (68.3 ± 22.0)	816 (67.8 ± 22.3)	128 (66.5 ± 25.4)	.63
5 y	1394 (71.2 ± 22.5)	678 (70.8 ± 22.9)	98 (72.5 ± 25.5)	.77

a Data are reported as n (mean ± SD). The covariates applied to the model (analysis of covariance) are age, sex (preoperative and postoperative KOOS), and medial meniscal resection (postoperative KOOS). ADL, Activities of Daily Living; KOOS, Knee injury and Osteoarthritis Outcome Score; QOL, Quality of Life; STS, side-to-side.

b Statistically significant.

### Revision ACLR

**Figure 1.**
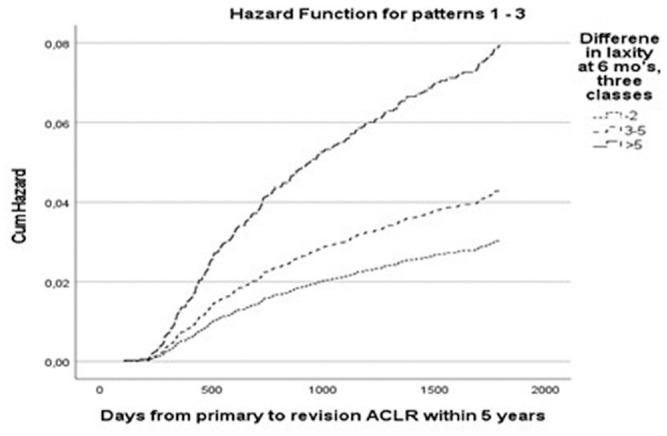
Cumulative (Cum) hazard of revision anterior cruciate ligament reconstruction (ACLR) based on Cox proportional hazards models (adjusted for age, sex, time from injury to primary ACLR, and medial meniscal resection).

The rates of revision ACLR within 5 years of primary surgery were as follows: STS ≤2 mm, 3.8% (116/3015); STS of 3 to 5 mm, 6.6% (95/1446); and STS >5 mm, 11.4% (27/236). The hazard for revision ACLR within 5 years of primary surgery was significantly higher for the groups with STS of 3 to 5 mm and STS >5 mm compared with the group with STS ≤2 mm (Table 3). The cumulative hazard of revision ACLR in the 3 laxity groups is shown in Figure 1.

**Table 3:** Relationships Between Postoperative STS Laxity and Revision ACLR Within 5 Years From Primary Surgery^*a*^

	HR (95% CI)	*P*
STS ≤2 mm	1 (reference group)	
STS 3-5 mm	1.42 (1.07-1.87)	.01^*b*^
STS >5 mm	2.61 (1.69-4.03)	<.001^*b*^

a The covariates applied to the model (Cox regression analysis) are age, sex, time from injury to primary ACLR, and medial meniscal resection. ACLR, anterior cruciate ligament reconstruction; HR, hazard ratio; STS, side-to-side.

b Statistically significant.

## Discussion

The most important finding of the present study was that a higher grade of knee laxity 6 months after ACLR was associated with an increased hazard of revision ACLR. The rates of revision ACLR within 5 years of primary surgery were 3.8%, 6.6%, and 11.4% among patients with STS ≤2 mm, 3 to 5 mm, and >5 mm, respectively. Patients with STS of 3 to 5 mm and >5 mm had significantly increased hazards of revision ACLR (HR, 1.42; 95% CI, 1.07-1.87; *P* = .01 and HR, 2.61; 95% CI, 1.69-4.03; *P* < .001, respectively) compared with patients with STS ≤2 mm. On the other hand, knee laxity was not associated with the subjective knee outcome after ACLR. The only significant difference between the groups was seen in the KOOS Symptoms subscale at the 1-year follow-up. However, this difference was numerically small and not clinically relevant.

Previously published studies investigating the relationship between postoperative arthrometric knee laxity and subjective knee outcome after ACLR have reported contrasting results. In line with the results of the present study, Cristiani et al^12^ found no correlation between 6-month KT-1000 arthrometer measurements and 1-year postoperative KOOS and the Lysholm score. Similarly, Tyler et al^40^ found no association between KT-1000 arthrometer measurements and Lysholm and Tegner scores at 1-year follow-up. Goodwillie et al^18^ found no differences in clinical outcome scores (KOOS, IKDC, Lysholm, and Tegner) between tight (STS ≤2 mm) and loose grafts (STS >5 mm) at a long-term (16-year) follow-up. In a similar study, Sundemo et al^35^ found no association between early (2-year) KT-1000 arthrometer measurements and long-term (16-year) subjective knee outcome (IKDC and Lysholm scores). The authors, however, applied mean STS differences, not stratified STS laxity, as in the present study. In contrast to the results of these studies, Sernert et al^34^ found a significant correlation between KT-1000 arthrometer measurements and the Lysholm score, and Magnussen et al^27^ found that patients with STS >6 mm 2 years after ACLR had inferior KOOS values and IKDC scores at a 6-year follow-up. In a recent large cohort registry study, Fiil et al^16^ found that patients with STS of 3 to 5 mm and >5 mm at 1-year follow-up had lower values in the KOOS Sport and Recreation and Quality of Life subscales compared with patients with STS ≤2 mm. Finally, Lindanger et al^24^ found that a slightly loose graft (STS 3-5 mm) at 6 months after ACLR was associated with an inferior Lysholm score compared with a tight graft (STS ≤2 mm) at a 25-year follow-up. Given the inconsistent findings present in the literature, it is difficult to draw any definitive conclusion about the association between arthrometric knee laxity measurements and subjective knee outcome. It should also be noted that comparisons between the studies are difficult to make because of differences in surgical techniques, graft choice, testing devices (KT-1000 arthrometer vs Rolimeter), anterior tibial loads applied with the KT-1000 arthrometer (89 N vs 134 N vs manual-max), patient samples, and different follow-up times. In the literature, rotational laxity has been more consistently associated with subjective knee outcomes.^4,22,35^

Interestingly, a high grade of postoperative (6-month) knee laxity (STS 3-5 and >5 mm) was associated with an increased hazard of revision ACLR within 5 years of the primary surgery. The results of the present study are in contrast to those of Goodwillie et al,^18^ who found that postoperative laxity >5 mm in the early postoperative period (6, 12, and 24 months postoperatively) was not associated with additional surgical procedures, including revision ACLR, at a 16-year follow-up. On the other hand, Lindanger et al^24^ found that an STS of 3 to 5 mm at 6 months after ACLR increased the risk of later (25-year follow-up) revision surgery. In a recent large cohort study from the Danish Knee Ligament Reconstruction Registry, Fiil et al^16^ demonstrated that a high level of knee laxity (STS 3-5 and >5 mm) 1 year after ACLR was associated with significantly higher revision ACLR rates within 2 years of the primary surgery. However, it should be noted that patients with STS >5 mm were advised to undergo revision ACLR because of the findings of high laxity, and patients with STS of 3 to 5 mm were advised to avoid activities, such as contact sports, with a high risk of ACL injury. This could have biased the results. In the present study, no advice was given to the patients to avoid particular physical activities or to undergo revision ACLR irrespective of the degree of arthrometric knee laxity measured at the 6-month follow-up. It might be hypothesized that a higher grade of knee laxity could make the knee more vulnerable to new traumas or could be responsible for a greater stress on the ACL graft itself, which is inclined to failure.^12^ At the same time, it is also possible that patients with a higher grade of postoperative knee laxity may have recurrent symptoms of instability, which may lead to revision ACLR.

Finally, it is interesting to note the progressive increased rate of medial meniscal resection in the laxity groups. The group with the highest grade of postoperative laxity (STS >5 mm) had the highest rate (29.2%) of medial meniscal resection. This finding confirms the essential role of the medial meniscus as a restraint of anterior tibial translation.^7,11,28,30^

Revision surgery is an important outcome parameter after ACLR. The present study highlights the importance of reestablishing normal knee laxity during ACLR to reduce the risk of future revision surgery. Surgeons should strive to meticulously diagnose and eventually address all knee injuries (meniscal injuries, including ramp lesions and root tears, and injuries to the anterolateral structures) that may be associated with increased knee laxity after ACLR.

The main strength of this study was the analysis of a large cohort (4697 patients). This enabled a detailed investigation of the differences in subjective knee outcome and the hazard of revision ACLR between the laxity groups. It also allowed a high statistical power and made the results generalizable. The patients underwent surgery at the same institution and followed a standardized rehabilitation protocol, which makes this study different from previous large cohort registry studies in which patients underwent surgery at different clinics with different surgical techniques and different grafts and followed different rehabilitation protocols. In the present study, we used arthrometric knee laxity measurements, which are considered to be more precise than manual knee laxity measurements.^13,41^ Another strength was that patients with concomitant knee ligament injuries were excluded, as these may have affected the outcomes of primary ACLR.^3,29^ In addition, only hamstring tendon ACLRs were included; therefore, the potential effect of the graft on knee laxity, subjective knee outcome, and risk of revision surgery^7,8,12,17^ was accounted for. Finally, we were able (through the SNKLR) to identify patients who underwent revision ACLR anywhere in the country.

Several limitations are present. First, the possibility that the arthrometric evaluation may be subject to interobserver variability, as it can be affected by the examiner’s skills, is often discussed as a potential limitation of studies using KT-1000 arthrometer measurements. However, the following factors reduced the influence of this possible limitation: (1) the physical therapists who performed the arthrometric evaluations were experienced and had used the KT-1000 arthrometer daily; (2) a standardized tibial displacement (134 N) was used and multiple measurements (≥3 for each knee) were performed; and (3) the study sample was very large. Second, we only studied the effect of arthrometric knee laxity on subjective knee outcome and the hazard of revision ACLR. The pivot-shift test is not registered in a standardized manner in our registry; therefore, any quantitative evaluation would have been difficult to perform. Moreover, it is known that the manual assessment of rotatory knee laxity varies greatly among examiners, as it is strongly affected by the examiner’s skill level.^2^ Third, the suboptimal 2-year (54.1%) and 5-year (46.4%) KOOS follow-up is another limitation. Loss to follow-up in patient-reported outcome measures is a known phenomenon of ACL registries, and these follow-up rates are comparable with those of previous studies based on large cohorts.^8,10,23^ However, the 2- and 5-year KOOS subscale values were still available for 2543 and 2181 patients, respectively. These samples gave a high statistical power and made the results generalizable. Another limitation is the lack of information regarding return to sport and postsurgery activity level. Differences in these outcomes between the laxity groups may have affected the hazard of revision ACLR. Finally, the outcome of the study was revision ACLR and not graft rupture. Our registry does not contain this information. We cannot, therefore, rule out differences in graft rupture between the laxity groups.

## Conclusion

A high grade of postoperative knee laxity (STS 3-5 mm and STS >5 mm) 6 months after primary ACLR was associated with an increased hazard of revision ACLR within 5 years, but it was not associated with an inferior subjective knee outcome.

## References

[1] Ageberg E Forssblad M Herbertsson P Roos EM . Sex differences in patient-reported outcomes after anterior cruciate ligament reconstruction: data from the Swedish knee ligament register. Am J Sports Med. 2010;38(7):1334-1342. 20410376 10.1177/0363546510361218

[2] Andersson D Samuelsson K Karlsson J . Treatment of anterior cruciate ligament injuries with special reference to surgical technique and rehabilitation: an assessment of randomized controlled trials. Arthroscopy. 2009;25(6):653-685. 19501297 10.1016/j.arthro.2009.04.066

[3] Bagherifard A Jabalameli M Ghaffari S , et al. Short to mid-term outcomes of single-stage reconstruction of multiligament knee injury. Arch Bone Jt Surg. 2019;7(4):346-353. 31448312 PMC6686067

[4] Bhardwaj A Solanki NS Jain H Raichandani K Raichandani S Daruwalla V . Comparison of outcome after ACL reconstruction in terms of subjective assessment of symptoms and function and clinical assessment of ligament stability. J Clin Orthop Trauma. 2018;9(2):172-174. 29896023 10.1016/j.jcot.2016.09.010 PMC5994999

[5] Butler DL Noyes FR Grood ES . Ligamentous restraints to anterior-posterior drawer in the human knee. A biomechanical study. J Bone Joint Surg Am. 1980;62(2):259-270. 7358757

[6] Cristiani R Forssblad M Edman G Eriksson K Stålman A . Age, time from injury to surgery and quadriceps strength affect the risk of revision surgery after primary ACL reconstruction. Knee Surg Sports Traumatol Arthrosc. 2021;29(12):4154-4162. 33661322 10.1007/s00167-021-06517-8 PMC8595184

[7] Cristiani R Forssblad M Engström B Edman G Stålman A . Risk factors for abnormal anteroposterior knee laxity after primary anterior cruciate ligament reconstruction. Arthroscopy. 2018;34(8):2478-2484. 29752059 10.1016/j.arthro.2018.03.038

[8] Cristiani R Mikkelsen C Edman G Forssblad M Engström B Stålman A . Age, gender, quadriceps strength and hop test performance are the most important factors affecting the achievement of a patient-acceptable symptom state after ACL reconstruction. Knee Surg Sports Traumatol Arthrosc. 2020;28(2):369-380. 31230125 10.1007/s00167-019-05576-2 PMC6994649

[9] Cristiani R Mikkelsen C Forssblad M Engström B Stålman A . Only one patient out of five achieves symmetrical knee function 6 months after primary anterior cruciate ligament reconstruction. Knee Surg Sports Traumatol Arthrosc. 2019;27(11):3461-3470. 30778627 10.1007/s00167-019-05396-4 PMC6800857

[10] Cristiani R Parling A Forssblad M Edman G Engström B Stålman A . Meniscus repair does not result in an inferior short-term outcome compared with meniscus resection: an analysis of 5,378 patients with primary anterior cruciate ligament reconstruction. Arthroscopy. 2020;36(4):1145-1153. 31811890 10.1016/j.arthro.2019.11.124

[11] Cristiani R Rönnblad E Engström B Forssblad M Stålman A . Medial meniscus resection increases and medial meniscus repair preserves anterior knee laxity: a cohort study of 4497 patients with primary anterior cruciate ligament reconstruction. Am J Sports Med. 2018;46(2):357-362. 29065270 10.1177/0363546517737054

[12] Cristiani R Sarakatsianos V Engström B Samuelsson K Forssblad M Stålman A . Increased knee laxity with hamstring tendon autograft compared to patellar tendon autograft: a cohort study of 5462 patients with primary anterior cruciate ligament reconstruction. Knee Surg Sports Traumatol Arthrosc. 2019;27(2):381-388. 29955930 10.1007/s00167-018-5029-9 PMC6394544

[13] Daniel DM Malcom LL Losse G Stone ML Sachs R Burks R . Instrumented measurement of anterior laxity of the knee. J Bone Joint Surg Am. 1985;67(5):720-726. 3997924

[14] Daniel DM Stone ML Sachs R Malcom L . Instrumented measurement of anterior knee laxity in patients with acute anterior cruciate ligament disruption. Am J Sports Med. 1985;13(6):401-417. 4073348 10.1177/036354658501300607

[15] Desai N Björnsson H Samuelsson K Karlsson J Forssblad M . Outcomes after ACL reconstruction with focus on older patients: results from the Swedish National Anterior Cruciate Ligament Register. Knee Surg Sports Traumatol Arthrosc. 2014;22(2):379-386. 24318509 10.1007/s00167-013-2803-6

[16] Fiil M Nielsen TG Lind M . A high level of knee laxity after anterior cruciate ligament reconstruction results in high revision rates. Knee Surg Sports Traumatol Arthrosc. 2022;30(10):3414-3421. 35333934 10.1007/s00167-022-06940-5

[17] Gifstad T Foss OA Engebretsen L , et al. Lower risk of revision with patellar tendon autografts compared with hamstring autografts: a registry study based on 45,998 primary ACL reconstructions in Scandinavia. Am J Sports Med. 2014;42(10):2319-2328. 25201444 10.1177/0363546514548164

[18] Goodwillie AD Shah SS McHugh MP Nicholas SJ . The effect of postoperative KT-1000 arthrometer score on long-term outcome after anterior cruciate ligament reconstruction. Am J Sports Med. 2017;45(7):1522-1528. 28277739 10.1177/0363546517690525

[19] Hefti F Müller W Jakob RP Stäubli HU . Evaluation of knee ligament injuries with the IKDC form. Knee Surg Sports Traumatol Arthrosc. 1993;1(3-4):226-234. 8536037 10.1007/BF01560215

[20] Jonsson H Kärrholm J Elmqvist LG . Laxity after cruciate ligament injury in 94 knees. The KT-1000 arthrometer versus roentgen stereophotogrammetry. Acta Orthop Scand. 1993;64(5):567-570. 8237326 10.3109/17453679308993694

[21] Kittl C El-Daou H Athwal KK , et al. The role of the anterolateral structures and the ACL in controlling laxity of the intact and ACL-deficient knee. Am J Sports Med. 2016;44(2):345-354. 26657572 10.1177/0363546515614312

[22] Kocher MS Steadman JR Briggs KK Sterett WI Hawkins RJ . Relationships between objective assessment of ligament stability and subjective assessment of symptoms and function after anterior cruciate ligament reconstruction. Am J Sports Med. 2004;32(3):629-634. 15090377 10.1177/0363546503261722

[23] Kvist J Kartus J Karlsson J Forssblad M . Results from the Swedish national anterior cruciate ligament register. Arthroscopy. 2014;30(7):803-810. 24746404 10.1016/j.arthro.2014.02.036

[24] Lindanger L Strand T Mølster AO Solheim E Inderhaug E . Effect of early residual laxity after anterior cruciate ligament reconstruction on long-term laxity, graft failure, return to sports, and subjective outcome at 25 years. Am J Sports Med. 2021;49(5):1227-1235. 33656379 10.1177/0363546521990801

[25] Liu SH Osti L Henry M Bocchi L . The diagnosis of acute complete tears of the anterior cruciate ligament. Comparison of MRI, arthrometry and clinical examination. J Bone Joint Surg Br. 1995;77(4):586-588. 7615603

[26] Lunde AS Lundeborg S Lettenstrom GS Thygesen L Huebner J . The person-number system of Sweden, Norway, Denmark, and Israel. Vital Health Stat. 1980;2(84):1-59. 7445446

[27] Magnussen RA Reinke EK Huston LJ , et al. Neither residual anterior knee laxity up to 6 mm nor a pivot glide predict patient-reported outcome scores or subsequent knee surgery between 2 and 6 years after ACL reconstruction. Am J Sports Med. 2021;49(10):2631-2637. 34269610 10.1177/03635465211025003 PMC9202674

[28] Musahl V Citak M O’Loughlin PF Choi D Bedi A Pearle AD . The effect of medial versus lateral meniscectomy on the stability of the anterior cruciate ligament-deficient knee. Am J Sports Med. 2010;38(8):1591-1597. 20530720 10.1177/0363546510364402

[29] Neri T Myat D Beach A Parker DA . Multiligament knee injury: injury patterns, outcomes, and gait analysis. Clin Sports Med. 2019;38(2):235-246. 30878046 10.1016/j.csm.2018.11.010

[30] Petrigliano FA Musahl V Suero EM Citak M Pearle AD . Effect of meniscal loss on knee stability after single-bundle anterior cruciate ligament reconstruction. Knee Surg Sports Traumatol Arthrosc. 2011;19(suppl 1):S86-S93. 21562842 10.1007/s00167-011-1537-6

[31] Robb C Kempshall P Getgood A , et al. Meniscal integrity predicts laxity of anterior cruciate ligament reconstruction. Knee Surg Sports Traumatol Arthrosc. 2015;23(12):3683-3690. 25217313 10.1007/s00167-014-3277-x

[32] Roos EM Lohmander LS . The Knee injury and Osteoarthritis Outcome Score (KOOS): from joint injury to osteoarthritis. Health Qual Life Outcomes. 2003;1:64. 14613558 10.1186/1477-7525-1-64 PMC280702

[33] Roos EM Roos HP Lohmander LS Ekdahl C Beynnon BD . Knee injury and Osteoarthritis Outcome Score (KOOS)—development of a self-administered outcome measure. J Orthop Sports Phys Ther. 1998;28(2):88-96. 9699158 10.2519/jospt.1998.28.2.88

[34] Sernert N Kartus J Köhler K Ejerhed L Brandsson S Karlsson J . Comparison of functional outcome after anterior cruciate ligament reconstruction resulting in low, normal and increased laxity. Scand J Med Sci Sports. 2002;12(1):47-53. 11985766 10.1034/j.1600-0838.2002.120109.x

[35] Sundemo D Sernert N Kartus J , et al. Increased postoperative manual knee laxity at 2 years results in inferior long-term subjective outcome after anterior cruciate ligament reconstruction. Am J Sports Med. 2018;46(11):2632-2645. 30067079 10.1177/0363546518786476

[36] Svantesson E Hamrin Senorski E Baldari A , et al. Factors associated with additional anterior cruciate ligament reconstruction and register comparison: a systematic review on the Scandinavian knee ligament registers. Br J Sports Med. 2019;53(7):418-425. 30018121 10.1136/bjsports-2017-098192

[37] Swedish National Knee Ligament Registry. Accessed May 5, 2024. http://www.aclregister.nu

[38] Tegner Y Lysholm J . Rating systems in the evaluation of knee ligaments injuries. Clin Orthop Relat Res 1985;98:43-49. 4028566

[39] Thomeé R Kaplan Y Kvist J , et al. Muscle strength and hop performance criteria prior to return to sports after ACL reconstruction. Knee Surg Sports Traumatol Arthrosc. 2011;19(11):1798-1805. 21932078 10.1007/s00167-011-1669-8

[40] Tyler TF McHugh MP Gleim GW Nicholas SJ . Association of KT-1000 measurements with clinical tests of knee stability 1 year following anterior cruciate ligament reconstruction. J Orthop Sports Phys Ther. 1999;29(9):540-545. 10518296 10.2519/jospt.1999.29.9.540

[41] Wiertsema SH van Hooff HJ Migchelsen LA Steultjens MP . Reliability of the KT1000 arthrometer and the Lachman test in patients with an ACL rupture. Knee. 2008;15(2):107-110. 18261913 10.1016/j.knee.2008.01.003

[42] Wroble RR Van Ginkel LA Grood ES Noyes FR Shaffer BL . Repeatability of the KT-1000 arthrometer in a normal population. Am J Sports Med. 1990;18(4):396-399. 2403189 10.1177/036354659001800411

